# The Fundoplication Revision of a Recurrent Large Paraesophageal Hiatal Hernia in a 63-Year-Old Female

**DOI:** 10.7759/cureus.87119

**Published:** 2025-07-01

**Authors:** Tristan Moseley, Walter M Sartor, Matthew D Overturf

**Affiliations:** 1 Medicine, Edward Via College of Osteopathic Medicine, Monroe, USA; 2 General Surgery, The Surgery Clinic of Northeast Louisiana, Monroe, USA; 3 Anatomical Sciences, Edward Via College of Osteopathic Medicine, Monroe, USA

**Keywords:** esophagogastroduodenoscopy (egd), gastroesophageal reflux disease (gerd), general surgery, laparoscopic toupet fundoplication, paraesophageal hiatal hernia

## Abstract

Paraesophageal hiatal hernias present significant clinical challenges due to their potential for incarceration and strangulation. The high risk of recurrence can also pose difficulties in management. We report a case of a 63-year-old Caucasian female with a history of recurrent paraesophageal hiatal hernia, initially diagnosed after a gastric incarceration event in 2014. Ten years later, imaging confirmed the recurrence of a large paraesophageal hernia containing the upper stomach. This report underscores the complexity of managing paraesophageal hiatal hernias, particularly recurrence following prior surgical interventions. It highlights the successful use of robotic-assisted revisional surgery in a complex case of recurrent paraesophageal hernia, thereby emphasizing both the rarity of the pathology and the value of advanced surgical approaches in managing challenging recurrences.

## Introduction

Hiatal hernia refers to a condition where a portion of the stomach protrudes upward through the diaphragmatic hiatus into the thoracic cavity. Disruption of the gastroesophageal junction (GEJ) can lead to reflux disease [1]. Hiatal hernias are classified into four types based on their location and contents: sliding (type I), paraesophageal (type II), mixed (type III), and massive (type IV). Understanding these classifications and anatomy is essential for accurate diagnosis and management.

Sliding hiatal hernia (type I) is the most common type of hiatal hernia, accounting for approximately 95% of cases. In this variant, both the GEJ and a portion of the stomach slide up through the hiatus into the thoracic cavity. This type is often associated with gastroesophageal reflux disease (GERD) due to the disruption of the lower esophageal sphincter (LES). Paraesophageal hiatal hernias (type II) are much less common, representing about 5% of all hiatal hernias. In this type, the GEJ remains in its usual position, while a part of the stomach herniates alongside the esophagus through the hiatus. Type II hernias are prone to incarceration or strangulation, which may lead to severe complications such as ischemia and perforation [2]. Mixed or compound hiatal hernias combine features of both sliding and paraesophageal hernias. In this type, the gastroesophageal junction and a portion of the stomach slide into the thoracic cavity, with additional parts of the stomach possibly herniating alongside the esophagus. Mixed hiatal hernias (type III) are relatively rare and may present with a combination of symptoms associated with both types. Massive hiatal hernias (type IV) involve the herniation of the stomach and other abdominal organs, such as the spleen or colon, into the thoracic cavity. It is extremely rare and can be linked to significant complications due to the displacement of multiple organs [3].

Paraesophageal hiatal hernias, although less common than sliding hernias, present a unique clinical challenge. Their rarity can result in diagnostic delays or misdiagnosis, as symptoms may be attributed to more prevalent conditions. The clinical presentation varies, with patients frequently experiencing severe symptoms such as chest pain, difficulty swallowing, and regurgitation. Complications like incarceration and strangulation pose critical concerns, requiring prompt surgical intervention to prevent serious outcomes. This case report illustrates the challenges of diagnosing and managing paraesophageal hiatal hernias, especially regarding recurrent hernias and postoperative complications. The relatively rare nature of this subtype underscores the need for thorough evaluation and personalized treatment planning to prevent potential negative outcomes and enhance the patient’s quality of life.

## Case presentation

A 63-year-old Caucasian female presented with a long history of recurrence of a previously corrected hiatal hernia. In 2014, she had experienced acute incarceration of the hiatal hernia, which had led to her hospitalization. The suspected gastric volvulus improved with endoscopic intervention, leading to the initial diagnosis of a hiatal hernia. In 2015, she had undergone laparoscopic hiatal hernia repair, and a partial fundoplication had been performed. Although she had experienced some relief, she had eventually begun to have symptoms again and required multiple esophagogastroduodenoscopies (EGDs) with esophageal dilations over the following years. Recurrence had not been visualized on EGD until 2023. At that time, she had deferred undergoing surgery due to insurance issues but had been closely monitored.

**Figure 1:**
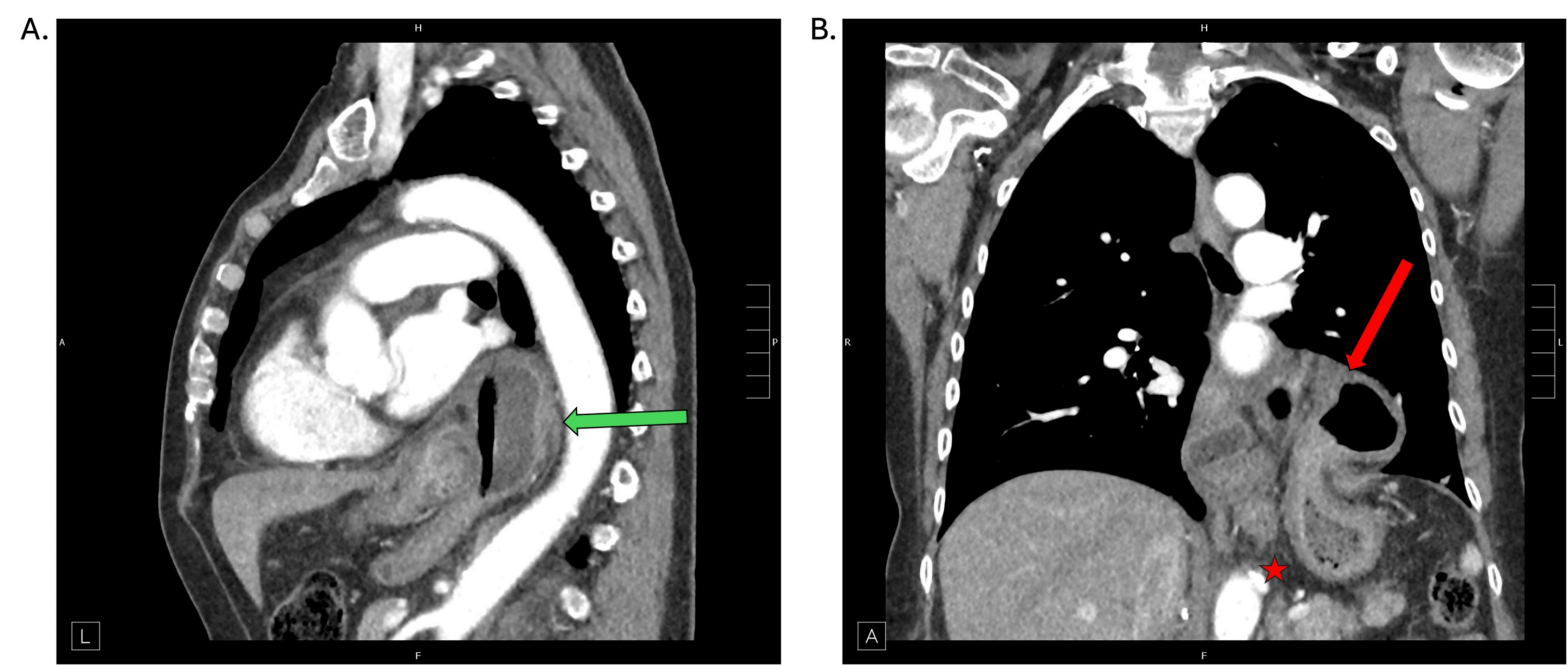
Thoracoabdominal CT scan Thoracoabdominal CT scan with sagittal (A) and coronal (B) views. Hernia sac above the diaphragm is seen in sagittal view (green arrow). The gastric fundus (red arrow) is superior to the preserved gastroesophageal junction (red star) CT: computed tomography

In 2024, the patient experienced a hip fracture that required urgent hip surgery. Postoperatively, she suffered from significant nausea and retching due to pain medications, and her previous hernia symptoms continued to worsen. She developed persistent heartburn, regurgitation, difficulty swallowing, and a dry cough, although there were no signs of bronchitis or pneumonia to suggest aspiration. As she endured severe symptoms with difficulty eating, stemming from postprandial chest pain and pressure, an EGD was initially performed for symptom management (e.g., dilation), which also confirmed the presence of the herniated stomach above the lower esophageal sphincter. An upper GI series was also used to evaluate esophageal anatomy, assess for reflux, and investigate possible dysmotility. In addition, a CT chest scan (Figure 1) was obtained for a detailed anatomic visualization of the hernia’s size and position. All three imaging studies confirmed the recurrence of the large paraesophageal hernia containing the upper half of the stomach. Before imaging confirmation, differential diagnoses included reflux disease, esophageal stricture, and esophageal dysmotility, which these modalities helped distinguish.

The patient underwent a semi-elective robotic-assisted Toupet fundoplication revision to address the recurrent hernia (a detailed, step-by-step description of the full procedure is provided in the Appendices). Intraoperatively, the hernia sac was found to be grossly adhered to the left parietal pleura, and extensive scarring in the mediastinum was noted. She tolerated the surgery well but required a chest tube due to a pneumothorax that developed during the dissection of the extensive scarring. Her postoperative course was otherwise uneventful, with successful resolution of the pneumothorax and subsequent removal of the chest tube. Her diet was advanced in a standard fashion, beginning with clear liquids on hospital day one and progressing to soft foods as tolerated. She was discharged on postoperative day two based on her meeting the following criteria: adequate pain control, tolerating oral intake, ambulating independently, afebrile status, appropriate bowel and bladder function, and radiographic resolution of the pneumothorax. During her two-week follow-up, she was doing well and tolerating soft foods. She was able to belch and have bowel movements. She reported no reflux, nausea, dysphagia, shortness of breath, or chest pain. The patient was no longer reliant on proton pump inhibitors for reflux control.

## Discussion

Paraesophageal hernias can occur with a gastric volvulus due to laxity in the stomach's peritoneal attachments and subsequent rotation of the gastric fundus. Such severe presentations are considered surgical emergencies. Current recommendations advocate for operative repair of all symptomatic paraesophageal hernias and completely asymptomatic large hernias in patients younger than 60 years and otherwise healthy [4]. The complexity of the repair is affected by the degree of herniation, the presence of adhesions, and other patient-specific factors. Advanced surgical techniques, such as robotic-assisted fundoplication, provide precise control and may improve outcomes for patients with recurrent or complicated paraesophageal hernias [5].

Several controversies surround the surgical management of recurrent paraesophageal hiatal hernias. The primary topics of debate include the effectiveness of crural mesh reinforcement during fundoplication and Roux-en-Y gastric bypass (RYGB) conversion in obese patients compared to redo fundoplication for recurrent hiatal hernias. Recurrence rates can reach up to 48% in patients with initial hernia sizes >5 cm, whereas smaller hernias (<5 cm) are associated with recurrence rates <25% at three years. Mean recurrence rates of approximately 20% have been reported following either Toupet or Nissen fundoplication for type II hernias. Recognized risk factors include a large initial hernia defect, obesity, tissue fragility, increased intraabdominal pressure from straining, coughing, or retching, and prior surgical manipulation [6]. Many cases of this nature are approached based on the patient's individual circumstances and the managing surgeon's preferred techniques.

According to the Society of American Gastrointestinal and Endoscopic Surgeons (SAGES) guidelines, laparoscopic fundoplication is the current standard procedure for paraesophageal hernias. Typically, a Nissen fundoplication (360°) is performed following most hiatal hernia repairs, unless there is evidence of esophageal dysmotility, in which case the Toupet (partial) fundoplication (270°) is the preferred choice. Compared to traditional open repair, minimally invasive laparoscopic techniques have resulted in better outcomes, shorter hospital stays, and faster recovery times [7].

Recent literature endorses the safety and efficacy of redo fundoplication in appropriately selected patients, despite the technical challenges associated with reoperative foregut surgery. A systematic review and meta-analysis by Schlottmann et al., involving over 2,000 patients across 30 studies, evaluated outcomes following laparoscopic redo fundoplication. The authors found that reoperation was associated with a relatively low rate of conversion to open surgery (6%) and a major morbidity rate of approximately 5%, suggesting that minimally invasive techniques remain feasible even in the setting of prior surgical intervention. Of note, nearly 80% of patients experienced significant symptomatic improvement, and over 80% reported enhanced quality of life following revision. Recurrence rates were reported at just over 10%, indicating that with proper surgical planning and technique, redo fundoplication can offer durable symptom control. These findings reinforce the importance of surgeon experience and meticulous technique, particularly when navigating dense adhesions and altered anatomy in the reoperative field [8].

This report illustrates the challenges of managing recurrent paraesophageal hiatal hernias, especially in the setting of prior surgical interventions and additional postoperative complications. Recurrent hernias can lead to significant morbidity and necessitate careful surgical planning. Although the SAGES guidelines do not address the topic, employing robotic-assisted techniques may provide advantages in precision and recovery [9]. The resolution of symptoms post-surgery emphasizes the importance of timely and effective intervention in managing complex hernia cases. The foremost challenge involves assessing the risks we are willing to accept regarding the possibility of a revision. Currently, fundoplication procedures remain the primary treatment for paraesophageal hiatal hernias, although there is a risk of recurrence. The alternative use of biologic and synthetic mesh for crural repair has proven effective in some instances, but long-term benefits linked to recurrence have been shown to diminish over time, irrespective of mesh implementation [10,11]. This lack of conclusive data hinders any updates to the current guidelines, and decision-making largely relies on the expert opinion of the managing surgeon.

## Conclusions

Recurrent paraesophageal hiatal hernias can present significant challenges, particularly in patients with a prior surgical history and additional complications. Reoperative hiatal hernia surgery requires an experienced surgical team and individualized planning. Tailoring the surgical approach to the patient's history, anatomy, and risk profile is essential for achieving optimal outcomes in complex, recurrent cases. Robotic partial fundoplication was successful in the case we reported, highlighting its potential benefits in managing complex hernias. Continued monitoring, timely follow-up, and individualized treatment plans are crucial for optimizing patient outcomes.

## Appendices

Fundoplication revision: step-by-step approach

Once in the operating room, the patient was placed under general endotracheal anesthesia with muscle relaxation to facilitate pneumoperitoneum. She was positioned in reverse Trendelenburg. The abdomen was accessed using a Veress needle through a small incision at the left anterior axillary line along the costal margin, with CO₂ insufflation to 15 mmHg. An 8-mm robotic port was placed supraumbilically in the midline, approximately 15 cm inferior to the xiphoid. A 30° laparoscope was introduced. Subsequent ports were placed under direct visualization: right rectus 8-mm robotic port; left rectus port; and left anterior axillary port at the costal margin. A subxiphoid incision was made for the insertion of a Nathanson retractor, used to retract the left lobe of the liver anteriorly, exposing the esophageal hiatus and proximal stomach. After robotic docking, the following instruments were used: Port 1 - fenestrated bipolar grasper; Port 2 - camera; Port 3 - harmonic scalpel; and Port 4 - tip-up atraumatic grasper.

Intraoperative findings revealed extensive herniation of the stomach into the thoracic cavity, including the distal body and pylorus. The fundus remained subdiaphragmatic. Dense adhesions were noted between the hernia sac and the left parietal pleura, and prior mesh was identified along the hiatus.

Dissection began on the right side, using sharp and blunt techniques with judicious electrocautery to free the stomach and surrounding mesentery from adhesions and prior mesh. Special attention was given to protect the lesser curvature, caudate lobe of the liver, and inferior vena cava. Dissection was then carried anteriorly and leftward, mobilizing the hernia sac from the diaphragm and mesh. Entry into the posterior mediastinum allowed for the reduction of the hernia sac and stomach back into the abdomen. The posterior vagus nerve was identified and preserved.

The prior partial fundoplication was dissected and taken down using sharp dissection to avoid thermal injury. A red rubber catheter was placed around the esophagus and secured with a locking clip for gentle traction. The crura were reapproximated posteriorly using a running 0-nonabsorbable suture. No new mesh was used due to the presence of prior mesh and the surgeon’s preference, given concerns regarding additional foreign material and potential complications such as erosion or fibrosis in a densely scarred field. A new 270° Toupet fundoplication was constructed by wrapping the gastric fundus posteriorly around the esophagus and securing it with three 0-silk sutures on each side.

During the dissection, a small left-sided pleural violation occurred, and a 24 French chest tube was inserted under direct vision. Intraoperative fluoroscopy confirmed full lung expansion and correct tube placement. The procedure concluded without further complications. All instruments were removed, the robot was undocked, and the patient was extubated and transferred to recovery in stable condition.
